# Diagnosis of myocardial infarction in early presenters using 1-hour delta of high sensitive Troponin I: a pilot study

**DOI:** 10.1016/j.clinsp.2026.101090

**Published:** 2026-09-08

**Authors:** Mohammed A. Jeraiby, Johar M. Iqbal, Ibrahim Ahmed Robadi, Jobran M. Moshi, Mahmoud M. Habibullah, Sajad Ahmad Dar, Sarah J. Mobarki, Imtenan A. Oberi, Yara Ajeebi, Yazan Z. Omar, Luai Alhazmi

**Affiliations:** a Department of Basic Medical Science, College of Medicine, Jazan University, Jazan, Saudi Arabia; b Department of Medical Laboratory Technology, College of Nursing and Health Science, Jazan university, Jazan, Saudi Arabia; c Department of Nursing, College of Nursing and Health Sciences, Jazan University, Saudi Arabia; d College of medicine, Jazan university, Jazan, Saudi Arabia; e Department of Internal Medicine, College of medicine, Jazan university, Jazan, Saudi Arabia

**Keywords:** NSTEMI, Hs-cTnI, ACS, 1-Hour delta hs-cTnI, Rule-out and rule-In NSTEMI, Emergency care assessment

## Abstract

**Background:**

The accurate and timely diagnosis of Non-ST Elevation Myocardial Infarction (NSTEMI) in early presenter is important to reduce false negative cases. We aimed to discern the diagnostic cut-off of high-sensitive cardiac Troponin I (hs-cTnI) in early presenters who lack preexisting cardiac morbidity.

**Method:**

This is a record-based study in which we included data from 457 patients who presented to emergency care with symptoms suggestive of suspected Acute Coronary Syndrome (ACS). We calculated sensitivity and Negative Predictive Value (NPV) for rule out and specificity and Positive Predictive Value (PPV) to rule in NSTEMI at various cut-off levels of hs-cTnI. In addition, we used binary logistic regression to identify the best predictor for NSTEMI diagnosis.

**Result:**

We observed 91% sensitivity and 79% NPV at a baseline hs-cTnI level below 2 ng/L. However, this was insufficient to rule out NSTEMI. In addition, we found 1-hour delta (absolute delta) hs-cTnI to be the best predictor of NSTEMI. To rule in NSTEMI, specificity was 95%, and PPV was 77% at 4 ng/L 1-hour delta hs-cTnI. The Area Under Curve (AUC) in the Receiver Operating Characteristic (ROC) was 0.796 (p-value = 0.001) for 1-hour delta hs-cTnI.

**Conclusion:**

In early presenters without prior heart disease, a single baseline hs-cTnI measurement below 3 ng/L provided 86% NPV, but this was insufficient for safe rule-out, confirming that serial sampling is necessary. Standard 99th percentile cut-offs performed poorly in this population. Incorporation of a 1-hour delta assessment improved diagnostic discrimination. Further, even a low 1-hour delta hs-cTnI (4 ng/L) can rule in NSTEMI. However, due to nature of this pilot study, our 4 ng/L rule-in criterion needs to be validated in further studies before being used in the clinics.

## Highlights

• Early hscTrop I levels evaluated for NSTEMI diagnosis in non-CAD early presenters.
• In this pilot study, a 1-hour absolute delta hs-cTnI of 4 ng/L showed 95% specificity for NSTEMI, though this finding requres validation.
• Baseline hscTrop I < 3 ng/L achieved 86% NPV for ruling out early NSTEMI.
• Delta hscTrop I was the strongest predictor with an AUC of 0.796.
• Standard cut-offs may miss early NSTEMI in patients without prior heart disease.

## Introduction

Acute coronary syndrome is the most common cardiac emergency worldwide.^1^ It happened due to a sudden stoppage of blood supply to the myocardium. Acute Myocardial Infarction (AMI) ensues when necrosis occurs due to this blockage of blood flow. During triage in the emergency room, AMI can be stratified based on electrocardiographic findings of the presence (ST-Elevation Myocardial Infarction; STEMI) or absence (Non-ST-Elevation Myocardial Infarction; NSTEMI) of ST-segment elevation. Though STEMI can be ruled out by the absence of ST-elevation in an Electrocardiogram (ECG), the NSTEMI needs an additional criterion to ascertain myocardial necrosis to differentiate from Unstable Angina (USA). The absence of prompt and accurate ruling out of NSTEMI may result in overcrowding in emergency departments, which can increase the adverse outcomes among patients regardless of their diagnosis.^2^^,^^3^ Thus, safe and rapid exclusion of NSTEMI is indispensable for triaging in the emergency department. This task is mainly accomplished by high-sensitive cardiac Troponins (hs-cTn).

Since the discovery of cardiac troponins, their understanding related to AMI has enormously increased in the last six decades.^4^ Despite an improved sensitivity, a single baseline level of hs-cTnI may not be sufficient to safely rule out NSTEMI. To address the challenge, serial estimation of hs-cTn was put in practice. This led to the development of various algorithms [0/1 h (hr), 0/2hr, 0/3hr] that are currently utilized for an efficient triaging of acute chest pain in emergency department (ED). However, despite the proven diagnostic superiority and cost-effectiveness of these rapid ED protocols, clinical adherence remains suboptimal.^5^

Currently, a baseline value more than the 99th percentile of high-sensitive cardiac troponin I (hs-cTnI) and a relative rise/fall in its level is recommended to diagnose NSTEMI. However, this practice met with challenges in patients presenting early to emergency care.^6^, ^7^, ^8^ Recently, Lowry et al. mentioned that the 99th percentile cut-off should not be used in early presenters to rule out NSTEMI.^9^ In addition, they provide evidence in support of a level below the limit of detection to safely rule out AMI among those patients.^9^ Moreover, a multitude of studies emphasized the low circulating levels of hs-cTnI in subjects with no prior history of coronary artery disease (CAD) or AMI.^10^^,^^11^ This may further complicate the diagnosis of NSTEMI among early presenters who lack CAD.

To answer these pertaining questions, we designed this pilot study to evaluate the efficacy of ESC 0/1hr algorithm in diagnosing NSTEMI among early presenters lacking CAD. In addition, we aimed to validate the existing standard baseline cut-off to rule out NSTEMI in those patients.

## Methods

We performed this retrospective cross-sectional study by recollecting the records of patients who presented with the symptoms of suspected ACS from July 2022 to September 2024 at the emergency department of King Fahad Central Hospital, Jizan, KSA. This pilot study is intended to generate hypotheses and provide preliminary estimates to inform larger, definitive studies, rather than to establish practice-changing cut-offs. A total of 457 eligible patients were included in the analysis. This study was approved by the Jazan ethics committee (Approval Number ‒ 2287). Due to the retrospective nature of the study and the use of anonymized patient data, the requirement for informed consent was waived by the Ethics Committee. The primary outcome was the confirmed diagnosis of NSTEMI. The AMI was defined according to the universal definition of myocardial infarction.^12^ In the absence of ST-segment elevation on ECG, NSTEMI was diagnosed clinically using the standard criteria.^13^ Briefly, patients presented with the symptoms of acute cardiac ischemia with non-ST-segment elevation on ECG, including transient or persistent ST-segment depression, and/or T-wave abnormalities, e.g., biphasic or inverted T-wave, were undergone hourly hs-cTnI quantification. A rise and/or fall of more than 20% from the baseline hs-cTnI levels or levels above the gender-wise upper reference limit of 99th percentile (Male – 34 ng/L; Female – 16 ng/L) at either time point (baseline or after 1 hour) were considered for echocardiographic evaluations. Patients with any unexplained regional wall motion abnormality on echocardiography were considered for the diagnosis of NSTEMI. In addition, all confirmed cases of NSTEMI underwent invasive coronary angiogram within 72 hours of the index event.

Patients with STEMI, non-cardiac chest pain, history of previous MI (or established diagnosis of CAD) and in which baseline hs-cTnI quantified after 3-hours of the onset of chest pain were excluded. However, patients with modifiable risk, e.g., Diabetes Mellitus (DM), Hypertension (HTN), Chronic Kidney Disease (CKD) and smoking were included in the study. In addition, any record deficient in 1-hour hs-cTnI level was excluded. Although baseline, 1-hour, 2-hour and 3-hour hs-cTnI levels were estimated in the patient’s samples, we included only baseline and 1-hour hs-cTnI levels in patients who presented to the emergency department within 3-hours of acute chest pain. The hs-cTnI was estimated in the patient’s plasma using Alinity i from Abbot, which is based on the principle of Chemiluminescent Microparticle Immunoassays (CMIA), according to the manufacturer’s instructions. The Limit of Detection (LOD) was 1.7 ng/L and the % Coefficient of Variation (%CV) was less than or equal to 6%.

### Statistical analysis

The patient’s characteristics are presented as the median (Interquartile Range; IQR) for continuous variables and number (percentage; %) for categorical variables. Binary logistic regression was used to identify the most appropriate predictor of NSTEMI. The odds ratio at 95% Confidence Interval (95% CI) with their respective p-values are mentioned in the table. The Area Under Curve (AUC) was calculated using the Receiver Operating Characteristic (ROC) curve. The Chi-square and Mann-Whitney tests were used to estimate significance. The p-value < 0.05 was considered as significant. Microsoft Excel was used to enter the patient’s data. All analyses were performed on Statistical Package for Social Sciences (SPSS) for Windows software (version 22.0; SPSS Inc, Chicago).

## Result

Table 1 provides the demographic details of the patients included in this study. We included 457 patients who presented to the emergency department with acute chest pain and suspected acute AMI, excluding STEMI. Among the patients, 152 (33%) had NSTEMI with a median age of 55.5 (IQR = 32.2) years. Around 42% (n = 190) were females in total, with 58 having NSTEMI. In addition, we observed an insignificantly higher prevalence of NSTEMI among male-gender, 35% in comparison to 30% among females. The median baseline hs-cTnI level for non-AMI patients was 4.75 (8.4), which was nearly half when compared to NSTEMI subjects (median/IQR; 9.9/16.4). The 1-hour delta hs-cTnI median level was 32% higher among NSTEMI (1.4/3.55) patients in comparison to non-AMI (−0.45/2.9).

**Table 1:** Baseline demographic details of the patients.

Particulars	Total (n = 457)		Non-AMI (n = 305)		NSTEMI (n = 152)	
	Median	IQR	Median	IQR	Median	IQR
Age^b^	52.7 ± 17.4		52.6 ± 17.5		52.9 ± 17.6	
Female^a^	190	41.6	132	43.3	58	38.1
Male^a^	267	58.4	173	56.7	94	61.8
BMI	25.8	3.4	25.6	3.3	26.1	3.3
Baseline	6.25	10.5	4.75	8.4	9.9	16.4
1-hour	7.1	12.7	4.2	9.4	11.7	13.7
Delta	0.3	2.8	−0.45	2.9	1.4	3.55
Relative Delta	4.7	44.9	−5.1	52.4	15.7	29.8
	**N**	**Percentage**	**N**	**Percentage**	**N**	**Percentage**
DM^a^	133	29.1	88	28.8	45	29.6
Hypertension^a^	166	36.3	98	32.3	68	44.7
CKD^a^	28	6.1	19	6.2	9	5.9
Smoking^a^	105	22.9	93	30.5	12	7.9

Values are presented as either median (IQR) or.

a N, Number (Percentage, %).

b Values are presented as Mean ± SD.

To rule out acute AMI, various cut-off levels of hs-cTnI were used. The 2 ng/L cut-off level yielded a sensitivity of 91% (95% Confidence Interval [95% CI 82–100]) and NPV of 79% (95% CI 57–100). However, the highest NPV observed at 3 ng/L was 86% (95% CI 74–99), with a corresponding sensitivity of 88% (95% CI 77–99). Interestingly, the 27 ng/L cut-off resulted in a poor sensitivity of 18% (95% CI 5–30), though the NPV was 67% (95% CI 57–77). A similar trend was observed with only 15% (95% CI 3–27) sensitivity and 65% (95% CI 55–76) NPV when gender-specific cut-offs [< 34 (M) < 16 (F)] of hs-cTnI levels were employed (Table 2). To ascertain the predictability of baseline, 1-hour, delta and relative delta hs-cTnI in ruling in AMI, we performed binomial logistic regression analysis. As presented in Table 3, delta hs-cTnI turned out as the most appropriate predictor for ruling in AMI with an (OR = 1.54, 95% CI 1.23–1.91, p = 0.001). Subsequently, we got (OR = 1.02, 95% CI 1.01–1.03, p = 0.003) as the odds ratio for the relative delta. On the contrary, both baseline and 1-hour hs-cTnI levels failed to predict AMI, significantly. Subsequently, we used the Receiver Operating Characteristic (ROC) curve to identify the AUC for various variables for ruling in the AMI, depicted in Table 4. We observed the maximum AUC, i.e., for the delta hs-cTnI 0.796 (95% CI 0.709–0.882, p < 0.001), followed by 1-hour hs-cTnI 0.735 (95% CI 0.632–0.838, p < 0.001). In addition, we found AUC for relative delta hs-cTnI 0.724 (95% CI 0.625–0.823, p < 0.001) and 0.672 (95% CI 0.560–0.784, p = 0.005) for the baseline hs-cTnI, respectively.

**Table 2:** Diagnostic performance of rule out cut-offs of hs-cTnI for NSTEMI.

Cut-off Levels	Sensitivity	Specificity	NPV	PPV
< 2 ng/L	91% (82 – 100)	18% (8 – 27)	79% (57 – 100)	38% (27 – 48)
< 3 ng/L	88% (77 – 99)	40% (28 – 52)	86% (74 – 99)	45% (33 – 57)
< 6 ng/L	71% (55 – 86)	58% (46 – 70)	78% (66 – 90)	48% (34 – 62)
< 27 ng/L	18% (5 – 30)	92% (85 – 99)	67% (57 – 77)	55% (25 – 84)
< 34 (M) < 16 (F)	15% (3 – 27)	89% (81 – 97)	65% (55 – 76)	42% (14 – 70)

Values are presented as percentage with their 95% Confidence Interval in parenthesis.

**Table 3:** Binary logistic regression of baseline, 1-hour, delta and relative delta for NSTEMI.

Variables	OR	Lower	Upper	p-value
Delta	1.537	1.235	1.913	0.001
Relative Delta	1.017	1.006	1.029	0.003
1-hour	1.012	0.99	1.033	0.285
Baseline	1.007	0.991	1.024	0.394

Values as presented as Odds Ratio (OR) at 95% Confidence Interval (95% CI).

**Table 4:** Area Under Curve (AUC) of baseline, 1-hour, delta and relative delta for NSTEMI.

Variables	AUC	Lower Bound	Upper Bound	p-value
Delta	0.796	0.709	0.882	0.001
Relative Delta	0.724	0.625	0.823	0.001
1-Hour	0.735	0.632	0.838	0.001
Baseline	0.672	0.56	0.784	0.005

Lastly, we used delta hs-cTnI as the most appropriate predictor for ruling in AMI. We used different cut-off levels of delta hs-cTnI for ruling in AMI. The specificity increased gradually as we increased the delta hs-cTnI values for ruing in AMI, as shown in Table 5. The highest specificity calculated at the level of 5 ng/L was 97% (95% CI 94 –100) with a PPV of 71% (95% CI 42–99). However, the PPV at this level was slightly lower than 4 ng/L 77% (95% CI 54–99), though with a comparable specificity, i.e., 95% (95% CI 90–100). This cut-off was identified through exploratory analysis of our dataset and requires confirmation in independent prospective cohorts before clinical application.

**Table 5:** Diagnostic performance of rule in cut-offs of hs-cTnI for NSTEMI.

Delta	Sensitivity	Specificity	NPV	PPV
0.5	74% (59 – 88)	68% (56 – 79)	82% (72 – 93)	56% (41 – 70)
1	62% (45 – 78)	74% (63 – 85)	78% (67 – 88)	57% (41 – 73)
1.5	53% (36 – 70)	81% (71 – 90)	76% (65 – 86)	60% (42 – 77)
2	44% (27 – 61)	85% (77 – 94)	74% (63 – 84)	63% (43 – 82)
3	38% (22 – 55)	90% (83 – 98)	73% (63 – 83)	68% (47 – 89)
4	29% (14 – 45)	95% (90 – 100)	71% (61 – 81)	77% (54 – 99)
5	15% (3 – 27)	97% (94 – 100)	67% (58 – 77)	71% (42 – 99)

Values are presented as percentage with their 95% Confidence Interval in parenthesis.

## Discussion

We investigated the utility of hs-cTnI in diagnosing NSTEMI in patients, without preexisting heart disease, who presented to the emergency department early with acute chest pain. The baseline and 1-hour hs-cTnI levels in a total of 457 patients, approached within 3-hours of their chest pain for institutional care, were used for the identification of cut-off with the highest specificity and PPV for ruling in NSTEMI. The diagnosis of NSTEMI in early presenters is a cumbersome task due to the lack of appreciable rise in hs-cTnI levels coupled with inconclusive ECG findings. Thus, a false negative rate is high, especially in patients undiagnosed of prior coronary artery disease. Recent reports suggest an increase in acute cardiac events among younger and middle-aged type 2 diabetic patients in the post-COVID-19 era.^14^^,^^15^ Therefore, our finding provides a valuable basis for further insight into the usefulness of hs-cTnI levels for the diagnosis of NSTEMI among early presenters.

Most of the subjects were male: 267 (58%) in our study, and 98 (21% of the total) of them were diagnosed with NTEMI. The median baseline (108%) and 1-hour (178%) hs-cTnI levels were higher among patients diagnosed with NSTEMI as compared to non-AMI. Further, a higher absolute rise in hs-cTnI was observed in NSTEMI patients after 1-hour. Our findings were supported by previous studies, though the exact levels might differ due to different sets of populations and types of patients included in the studies.^16^^,^^17^

While using standard cut-offs of baseline hs-cTnI, either overall 27 ng/L or gender-specific (< 34 for Males and < 16 Females), we failed to observe the required sensitivity (15% to 18%) and NPV (65% to 67%) to rule out AMI. However, 88% sensitivity and 86% NPV were seen when a 3 ng/L cut-off was used. This level is slightly higher than the limit of detection (LOD = 1.7 ng/L). Our findings are in contradiction of the previous findings.^18^^,^^19^ This could be due to the inclusion of only early presenters in our study. In addition, these reports did not mention the duration of chest pain of the patient to their baseline hs-cTnI levels. Thus, a meaningful comparison is out of context. However, recently, Lowry et al.^3^ mentioned higher sensitivity and NPV than our observation for baseline hs-cTnI in patients with acute chest pain of less than three hours duration at various cut-off levels including below limit of detection. This discrepancy may be due to the inclusion of a large number of patients (40%) with preexisting cardiac disease by Lowry et al. In addition, our study has a very small number of subjects (457) in comparison to them (41,103).^8^

Both absolute (delta) and relative (relative delta) changes in hs-cTnI had proven usefulness in the early diagnosis of NSTEMI, though with an unsettled debate.^20^ Recent studies suggested higher efficiency of absolute delta in ruling in AMI.^21^^,^^22^ Comparably, we provide here evidence that absolute delta is superior to relative delta and the baseline as well as 1-hour hs-cTnI levels in ruling in AMI. Simpson et al. used a minimum 2-hour delta and Kim et al. employed 3-hour delta, which is different from our study of 1-hour. Previously, Neumann et al. used a 1-hour delta to rule in AMI, though their study did not analyze early presenters or lack of cardiac comorbidity.^18^ Nonetheless, it is still contested which is superior, the absolute or relative delta hs-cTnI.^23^

Furthermore, the optimum cut-off in our study to rule in AMI came at a level of 4 ng/L of absolute delta with the highest PPV (77%) and specificity of 95%, which is comparable to 5 ng/L (97%). Our level of delta hs-cTnI was considerably lower than the standard recommendations of 14 ng/L^13^^,^^24^ and previous reports.^18^ Additionally, our 4 ng/L threshold on the Alinity i (Abbott) remains lower than the ≥ 6 ng/L 1-hour delta cut-off maintained by current ESC Guidelines for the Architect (Abbott) assay, though both these instruments based on the same assay principle.^25^ Notably, the wide CI for the PPV at 4 ng/L (54–99%) reflects statistical imprecision in this single-center pilot study with a modest sample size. Therefore, this finding should be interpreted cautiously and considered hypothesis-generating, requiring validation in larger prospective multicenter cohorts before clinical implementation. In addition, we obtained a significant (p-value = 0.001) AUC value of 0.796 in ROC curve for delta hs-cTnI, which is higher than AUC value for relative delta (AUC/p-value; 0.724/0.001). However, a significant 0.796 AUC value may not provide adequate discrimination to be used alone in the diagnosis of an event. Importantly, the diagnosis of NSTEMI in acute care lies on the symptomatology and ECG findings suggestive of acute myocardial ischemia coupled with significant myocardia injury supported by a rise and/or fall in the levels of cardiac troponins (cTn). Moreover, the troponin level is influenced by a variety of factors, namely age, gender, prior history of adverse cardiac events and duration of chest pain.^26^^–^^30^ The male gender,^28^ advanced age,^26^^,^^27^ and presence of cardiac comorbidity tend to have higher hs-cTnI.^9^^,^^10^ On the other hand, a lower level of hs-cTnI was observed in active smokers.^29^ In addition, troponin level rises after 4 to 6 h of AMI, followed by a peak in approximately 18 – 24 h.^30^ However, it can be detected as early as 90 min following myocardial injury and levels can further increase after 3 h of the onset of symptoms.^30^ Moreover, the troponin level corresponds with the size of the infarct, though more time is required to ascertain infarct size with hs-cTnI levels.^31^ We included 176 (38%) patients who were younger than 50 years and 105 (23%) current smokers in our study. In addition, we did not include previously diagnosed CAD patients, whereas 36% of them were hypertensive and 29% had type 2 diabetes mellitus. Furthermore, 28 patients with CKD (chronic kidney disease) were included in the study. Therefore, more than half of the study subjects were having risk factors for CAD. Lastly, since the rise and fall of cTn levels are based on time and we presented duration of chest pain in hours (not in minutes) due to retrospective nature of the study, we were unable to identify at which time, in minutes, the appreciable rise in cTn levels occurred in those patients diagnosed with NSTEMI.

Although we present important insights concerning the diagnostic ability of hs-cTnI for NSTEMI in non-CAD patients who are early presenters, this study has certain limitations. Firstly, the findings of our study cannot be generalized as we included only a specific subcategory of patients. Secondly, due to the nature of the study, it needs to be validated in a prospective cohort. Thirdly, it’s a single-center study using the Saudi population only. Therefore, multicentric study in diverse ethnicities is required to corroborate the outcome. Fourthly, this study includes small number of NSTEMI patients (n = 152), thus these findings need validation in large-scale studies. Fifthly, the requirement for echocardiographic wall motion abnormality as part of the diagnostic pathway may have selected for a subset of NSTEMI patients with more definite myocardial injury, potentially introducing selection bias. Sixthly, we acknowledge the limitation of documentation of duration of chest pain in hours. The cTn, being a time-sensitive biomarker, might have provided more valuable insight in minutes rather in hours. The future works in this regard might settle the issue. Seventhly, since a multitude of studies had provided the ethnic and regional variations in the levels of cTn, our 4 ng/L cut-off of delta hs-cTnI to rule in NSTEMI needs to be validated in a variety of setups including disparate ethnicities. Lastly, as with most studies of troponin in suspected ACS, our diagnostic standard incorporated troponin results as part of clinical decision-making. This reflects real-world practice but means our estimates reflect clinical diagnostic performance rather than accuracy against an independent gold standard.

## Conclusion

Our findings suggest that a single baseline hs-cTnI measurement may be insufficient to safely rule out NSTEMI in early presenters without prior CAD, and incorporation of a 1-hour delta assessment appears necessary for improved diagnostic accuracy. Thus, this may result in higher morbidity as well as mortality. In addition, a low absolute delta of hs-cTnI can rule in NSTEMI with high specificity. Lastly, we acknowledge that our study is a pilot study, and our findings needs to be validated in multiethnic and large multicentric studies before being employed.

## Authors’ contributions

MJ, JI, IR, JM, MH, and SD contributed to the conception and design of the study, performed the literature review, data analysis, and were involved in drafting the manuscript. SM, IO, YA, and YO were responsible for the acquisition of data and contributed to the data collection process. LA provided critical revision of the manuscript and approved the final version for submission.

## Funding

This research did not receive any specific grant from funding agencies in the public, commercial, or not-for-profit sectors.

## Data availability

The data that support the findings of this study are available from the corresponding author upon reasonable request.

## Declaration of competing interest

The authors declare no conflicts of interest.
